# Amyloid hybrid membranes for bacterial & genetic material removal from water and their anti-biofouling properties†

**DOI:** 10.1039/d0na00189a

**Published:** 2020-09-11

**Authors:** Archana Palika, Akram Rahimi, Sreenath Bolisetty, Stephan Handschin, Peter Fischer, Raffaele Mezzenga

**Affiliations:** ETH Zurich, Department of Health Sciences and Technology Schmelzbergstrasse 9 8092 Zurich Switzerland; BluAct Technologies GmbH Schmelzbergstrasse 9 8092 Zurich Switzerland; ETH Zurich Department of Materials Wolfgang-Pauli-Strasse 10 8093 Zurich Switzerland raffaele.mezzenga@hest.ethz.ch +41 44 632 9140 +41 44 632 1603

## Abstract

Water scarcity and contamination by biological pollutants are global challenges that significantly affect public health. Reverse osmosis, nanofiltration and ultrafiltration technologies are very effective for the elimination of pathogens and most contaminants but associated with considerable capital and operating costs, high energy consumption and the use of chlorinated chemicals to suppress membrane fouling. Additionally, the pressure needed by these techniques may disrupt the pathogenic microbial cell membranes, causing the release of genetic material (fragments of DNA, RNA and plasmids) into the water. Here, we introduce the simultaneous removal of both bacteria and associated genetic material using amyloid hybrid membranes, *via* a combined adsorption and size exclusion mechanism. Amyloid hybrid membranes can remove upto and beyond 99% of the genetic material by adsorption, where amyloid fibrils act as the primary adsorbing material. When the same membranes are surface-modified using chitosan, the anti-biofouling performance of the membranes improved significantly, with a bacterial removal efficiency exceeding 6 log.

## Abstract 2

Introducing the simultaneous removal of both bacteria and associated genetic material using amyloid hybrid membranes, *via* a combined adsorption and size exclusion mechanism.

## Graphical abstract

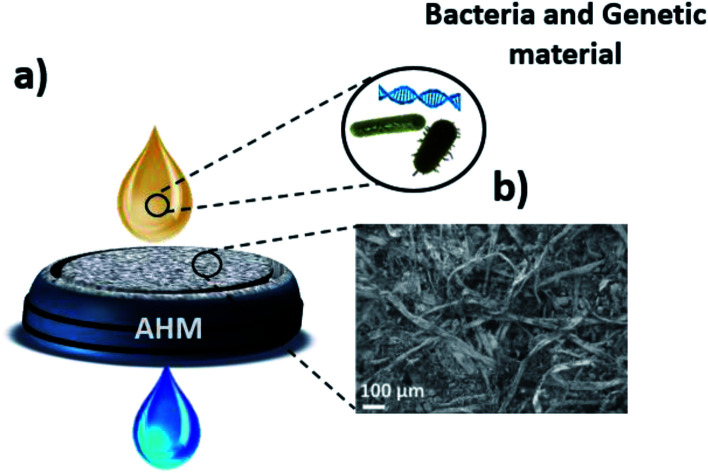

## 1. Introduction

Worldwide more than 1.2 billion people do not have access to safe drinking water, resulting in more than five million deaths each year.^1–3^ Biological contamination is still a major drinking water problem in many countries of the world.^4^ Management of microbiological risks in drinking water is crucial for public health protection.^5^ Although great efforts have been carried out, people worldwide still have to drink water contaminated by feces^6,7^ contributing to the spread of those pathogenic bacteria related to fecal contamination of water, such as *Escherichia coli* (*E. coli*), *Enterococcus faecalis*, *Salmonella* and *Clostridium perfringens*.^8,9^ Cholera, gastroenteritis, typhoid fever, bacillary dysentery and acute diarrhea are the most severe bacterial diseases transmitted through water.^10,11^*Legionella* is another highly prevalent bacterium that can grow in potable water systems, lakes and streams and can cause legionellosis.^12^ Annually 8000 to 18 000 cases of legionellosis diseases are reported in the USA.^13^ Monitoring the microbiological composition in drinking water is expensive and time-consuming so that it remains a major concern to preserve the quality of water supplies; the development of affordable technological solutions toward microbiologically safe drinking water remains a global challenge.^14^ Current methods for the removal of biological contamination from the water mostly rely on size exclusion techniques, such as ultrafiltration, nanofiltration and reverse osmosis,^15^ which are cost, energy and pressure intensive. Cost-effective disinfection methods such as chlorination, boiling, use of chemicals, ozonation and UV (ultra-violet) radiation^16^ have been developed and used for decades for efficient biological decontamination of water. However, none of these methods offers fully satisfying solutions. Pressure-driven membrane processes^17^ may disrupt the cell membrane of pathogenic bacteria^18–21^ due to high pressure and harsh conditions used during the processes, leading to the release of genetic material which could contain antibiotic-resistant genes (ARGs),^22,23^ (fragments of DNA, RNA and plasmids) into drinking water. Disinfection methods inactivate the pathogens but do not remove genetic material associated with them, *e.g.* the emerging contaminants (ARGs), which have become today a public concern.^24^ The spread of bacteria-related DNA could worsen the already growing problem of drug resistance among potentially infectious microbes.^25^ ARGs can be detected in a variety of environments such as hospital wastewater^26^ and wastewater treatment plants^27,28^ and can be transferred from human and animal sources to different environmental compartments, including drinking water sources, ultimately threatening human health.^29^ Therefore, their removal from drinking water is absolutely necessary, but due to their dimensions smaller than 1 nm, it is difficult to remove them in current water treatment processes, even *via* ultrafiltration, microfiltration and nanofiltration.^30^

Earlier studies revealed that amyloid hybrid membranes (AHM) may be able to remove bacteria by a size-exclusion mechanism,^31–34^ but we did not investigate this effect systematically, nor considered whether genetic material from biological contamination could be removed from water by the same membranes. This paper focuses on the removal efficiency of the amyloid hybrid membranes for both bacteria and genetic material by the systematic investigation. We evaluate the individual adsorption capacity of the hybrid membrane components, *i.e.*, cellulose, carbon and amyloid fibrils and we investigate the reusability of the membrane over several consecutive cycles. We finally quantify the efficiency for the removal of genetic material (prepared from *E. coli*) and bacteria by using the most common pathogenic bacteria in drinking water, *i.e. E. coli*, *Salmonella typhimurium* and *Legionella pneumophila*.^35,36^

Membrane biofouling which means biofilm formation over time is a major problem in the membrane filtration process.^37^ Microorganisms, including bacteria, are the main source of membrane biofouling.^38^ Biofilm formation is a slow multistage process, where microbial growth can take from a couple of weeks to several months.^39^ Yet, the initial step (adsorption of microbial cells) is relatively fast and typically occurs within the first two hours.^40^ The attachment of microbial cells on the surface is generally more favorable on hydrophobic and nonpolar surfaces. Bacteria produce extracellular polymeric substances (EPS), which anchor the cells to the substrate and stimulate further additional microbial colonization of the membrane surface.^41^ Membrane biofouling causes severe losses in the performance of membranes, particularly in flux. To avoid this problem, the membranes should be changed frequently, which further increases the cost of the filtration process. These problems are the main limitations for the application of the membrane process in industrial scale water treatments.^42^ Thus, emerging and cost-effective solutions to address this problem remain highly demanded.

As reported in previous works, surface hydrophilicity is a critical factor greatly enhancing the membrane anti-biofouling performance.^44^ Among diverse methods to improve the membrane hydrophilicity, the coating of the membrane surface with hydrophilic polymers has been regarded as an effective alternative.^43,44^ The advantage of this method compared to other methods such as surface grafting and polymer blending relies on its operation simplicity and suitability for large-scale applications.^45^

Here, we apply a surface coating polymer method^46^ to increase the biofouling resistance of the AHM. Among various hydrophilic polymers, chitosan is selected for its hydrophilicity, and the demonstrated capacity to mitigate the membrane biofouling.^45^ Numerous studies demonstrated that the presence of chitosan on the membrane surface is beneficial to increase hydrophilicity and therefore improve the fouling resistance of membranes.^47^ Chitosan is furthermore environmentally friendly, non-toxic, and has excellent antibacterial and hemostatic properties.^46^ Therefore, we selected chitosan to be used as an extra coating layer on the AHM meant to decrease the effect of biofouling.

## 2. Results and discussion

**Fig. 1:**
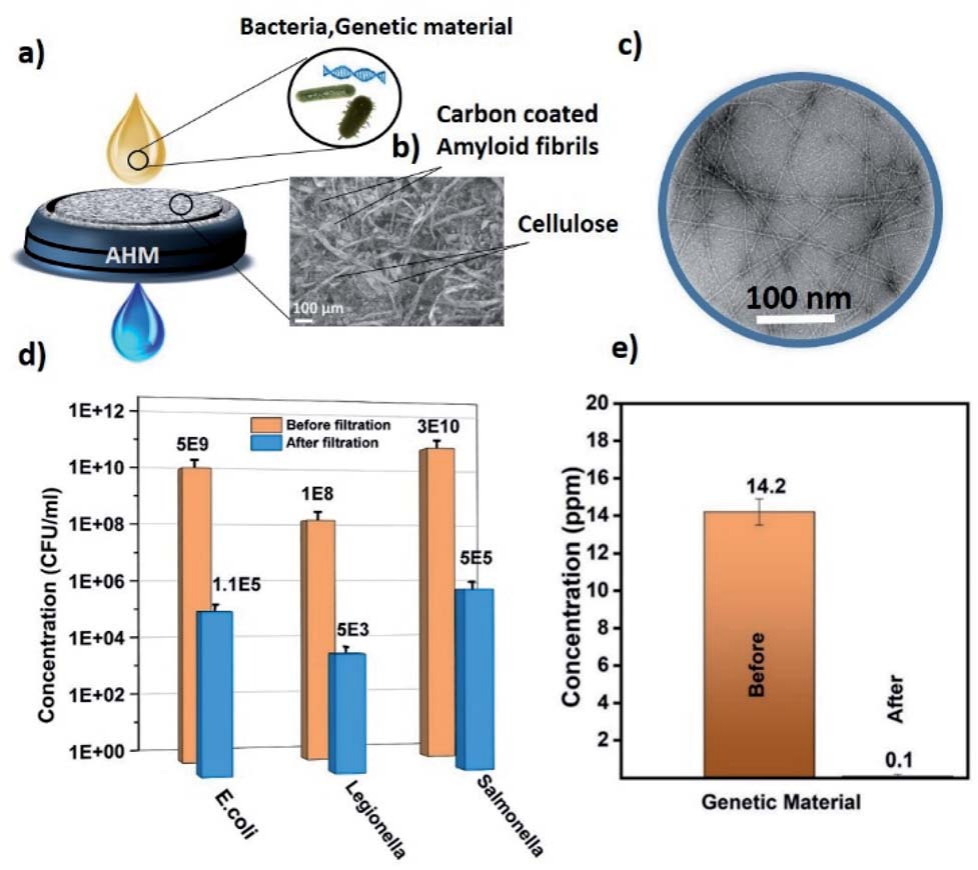
(a) Schematic representation of the AHM for the removal of bacteria and genetic material. (b) SEM image of the AHM. (c) TEM image of amyloid fibrils. (d) *E. coli*, *Salmonella* and *Legionella* bacteria concentration before and after filtration by AHM. (e) Genetic material concentration before and after filtration by the AHM.

Fig. 1a shows the schematic representation of the microbiological pollutant removal by the AHM. Due to the pore size of the membrane, bacteria cannot permeate through the membrane and removed from the water by a size exclusion process. The pore size of the AHM was measured using a gas–liquid porometer POROLUX100 (see ESI S3†). With respect to genetic material, the amyloid fibrils act as an adsorbent material *via* combined hydrophobic and hydrogen-bonding interactions.^48,49^ Both amyloids and siRNA and DNA possess multiple hydrogen bonding acceptor/donor moieties, therefore a strong hydrogen-bonding contribution is expected. In addition, hydrophobic and van der Waals interactions are also expected to contribute. Furthermore, the amyloid fibrils are ampholytic, therefore they possess both negative and positively charged groups, the latter capable to interact with the negatively charged genetic material.^50^Fig. 1b represents an SEM image of the surface of the hybrid membrane. It clearly shows the cellulose fibers and the activated carbon particles. The typical morphology of amyloid fibrils was studied by TEM and can be observed in Fig. 1c. Fig. 1d represents the *E. coli*, *Salmonella* and *Legionella bacteria* concentration before and after the filtration through the 2 wt% AHM. To detect the concentration of the bacterial cultures, serial dilutions were performed, plated on respective agar plates and incubated at 37 °C. Colony-forming units were measured to determine the CFU ml^−1^. Characterization details are provided in the ESI.† The measurements were performed in triplicates to get the average concentration values. The initial feed solution of the *E. coli* has a concentration of 5 × 10^9^ CFU ml^−1^, which is reduced to 1.1 × 10^5^ CFU ml^−1^ after the filtration, corresponding to an efficiency of 99.998%.

The initial concentration of the feed solution of *Legionella* is 1 × 10^8^ CFU ml^−1^ and after filtration, the concentration is decreased to 5 × 10^3^ CFU ml^−1^ having an efficiency of 99.9999%. In the case of *Salmonella*, the concentration is reduced from 3.8 × 10^10^ CFU ml^−1^ to 5 × 10^5^ CFU ml^−1^ after the filtration, corresponding to an efficiency of 99.998%. The different logarithmic removal efficiency can be explained by the size of the bacteria: the approximate size of the *E. coli* is 2 μm long by 0.5 μm wide,^51^*Salmonella* is 2 to 5 μm long by 0.5–1.5 μm wide^52^ and *Legionella* is 2 to 20 μm long by 0.3–0.9 μm wide.^53^ These results together indicate that the membrane operates with a 5 to 6 log reduction of the bacteria. This efficiency can be further improved by reducing the mesh size of the membrane, but the operation of the membrane filtration will need additional pressure for the removal mechanism to operate correctly. We are interested in membranes that can work by gravitational water flow, without any other energy or power required, so that the membranes can be used also in the household of remote areas, where no electrical energy is available. We show later below how efficiency can be further increased without significant compromise on the flow rate.

**Fig. 2:**
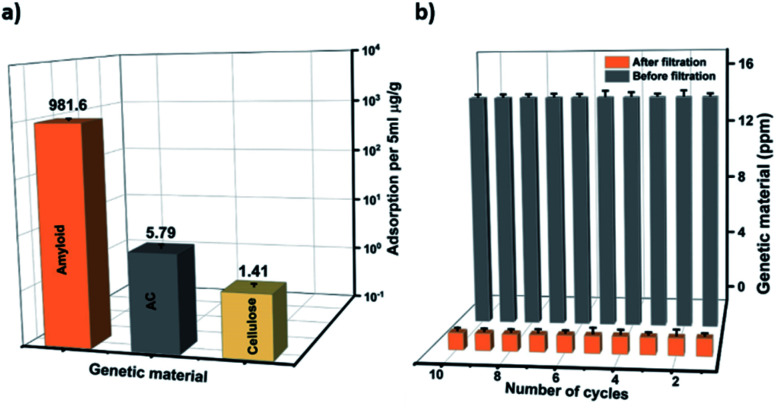
Performance of the AHM for genetic material removal. (a) Specific adsorption of the genetic material individually by the amyloid fibrils, activated carbon and cellulose. The filtration experiments were carried out at a pH 7 of the feeding solution (b) Genetic material removal after 10 cycles of filtration.

In order to test whether the composite membranes can remove genetic material released from bacteria, genetic material was filtered through the AHM. The concentration range was adjusted to stimulate the values of severely polluted water by genetic material. Fig. 1e depicts the concentration values of genetic material before and after filtration. The concentration of genetic material was measured using an absorption-based NanoDrop (Thermo scientific NanoDrop 2000). A solution containing a concentration of 14.2 ppm was filtered through the AHM and the concentration after filtration decreased to 0.1 ppm. This indicates that the membrane has a removal efficiency as high as 99.3%. Since the membrane has several components, we investigated the specific adsorption of individual components of the AHM (amyloid, cellulose and activated carbon). Membranes prepared with each individual component as mentioned in the Materials and methods section (ESI†) were used. The genetic material having a concentration of 14.2 ppm was passed through each membrane. The adsorption capacity was estimated for activated carbon, cellulose and amyloids, normalized by the amount of the adsorbing material (Fig. 2a). Activated carbon is well known for the adsorption of both the organic and biological pollutants,^54^ accordingly, the activated carbon removes 5.79 μg g^−1^ of the adsorbent, whereas cellulose adsorbs only 1.41 μg g^−1^. Amyloids, on the other hand, removed the genetic material up to 981.6 μg g^−1^. This indicates that the amyloid adsorption performance is more than two orders higher compared to activated carbon. Therefore, amyloid fibrils within the hybrid membrane, have the dominant role in removing the genetic material from water.

The reusability of the membrane was investigated by filtering 10 ml of genetic material (14.2 ppm) through 0.0002 m^2^ AHM in ten consecutive cycles. The concentration of the genetic material before and after the filtration during these ten consecutive cycles is shown in Fig. 2b. The results show that the hybrid membrane works very efficiently during these ten cycles of filtration.

**Fig. 3:**
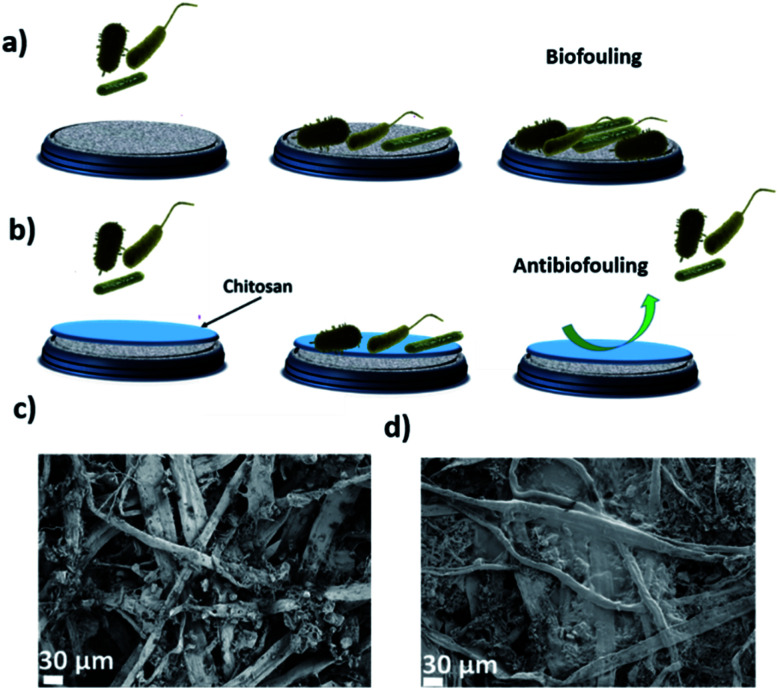
(a) Shows the schematic representation of the membranes biofouling with the bacteria and the genetic material. Our strategy to improve the anti-biofouling properties by surface modification of the AHM with the chitosan is schematically shown in (b). The comparison of the surface morphology for the pristine and the chitosan-coated AHM (CCAHM) was first studied by scanning electron microscopy (SEM). Cellulose fibers can be observed on the surface of the uncoated hybrid membrane (see (c)), while the smooth surface of the chitosan coating, covered some parts of the hybrid membrane (see (d)).

Adsorption isotherms of the membrane were determined by filtering genetic material through a 0.0002 m^2^ AHM membrane and the concentration of the adsorbed genetic material after filtration is measured by using a NanoDrop spectrophotometer. From the adsorption isotherm, 1200 g of the hybrid membrane can remove 124 g of the genetic material (see ESI, Fig. S1†). The excellent performance of the membrane in the removal of genetic material is understood from the binding of the genetic material with the amyloid fibrils in the membrane. The binding may be due to different interactions: possibly due to hydrophobic, van der Waals and electrostatic interactions among the positively charged groups of the ampholytic amyloids and the negative DNA. The above discussion and results indicate that AHMs are excellent candidates to remove the bacterial contamination and its genetic material from the water. However, the accumulation of these biological components on the membrane surface can cause biofouling which can further inhibit or slow down the flow rate of water through the membrane. To overcome this problem, the membrane surface is modified with chitosan and discussed below (Fig. 3).

**Fig. 4:**
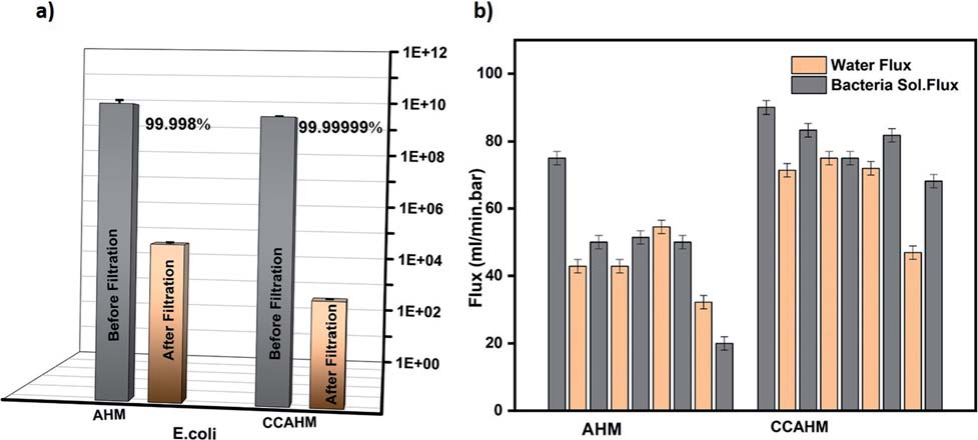
(a) Concentration of the *E. coli* before and after the filtration by the AHM and CCAHM membranes. (b) Water flux and bacteria solution flux during cyclic filtration for the pristine hybrid membrane and the chitosan-coated hybrid membrane.

The CCAHM showed improved bacteria rejection in comparison to the pristine membrane (Fig. 4a). The highly hydrophilic coating on the membrane surface helped to improve bacteria rejection from 99.998% for the pristine membrane to 99.99999% when tested against *E. coli*. The bacterial rejection is increased from 5 log to 7 log. High bacteria rejection may be attributed to two mechanisms including cell membrane disruption and size exclusion. From previous studies, it has been demonstrated that NH_2_ groups of chitosan can bind with the phospholipid bilayer in the bacterial cell membrane. This interaction disrupts the barrier properties of the bacterial outer membrane, resulting in the release of intracellular electrolytes such as potassium ions, glucose, and nucleic acid, *etc.*, thus leading to cell death.^55–57^ The other mechanism is a size exclusion effect, which can be illustrated by the fact that the chitosan coating led to the formation of a denser and thicker active layer resulting in the decrease of the pore size of the membrane, which improved the high rejection of the bacteria.

The removal of genetic material was also investigated with the CCAHM. In comparison to the AHM, CCAHM results in higher removal efficiency of the genetic material, possibly, chitosan also have ability to conjugate with the genetic material.^58^

In bio-fouling evaluation, the membrane performance is typically evaluated in terms of water flux and bacteria flux during cyclic filtration tests. The biofilm formation directly influences the flow rate of the membrane. Therefore we measured the changes in flow rate induced by the biofilm formation. The complete details of the relationship between the flux and biofilm formation are provided in the ESI.† The cyclic operations of the bacteria filtration are shown in Fig. 4b. Significant fouling was observed in cyclic filtration experiments with the pristine hybrid membrane, as indicated by an appreciable decline (75%) of the water flux from 75 ml min^−1^ bar^−1^ to 19 ml min^−1^ bar^−1^ after five cycles. The large flux reduction and the small flux recovery of the pristine membrane is the result of the biofilm formation by the interaction between the membrane surface and microorganisms. Since the CCAHM is more hydrophilic, the bacteria have a lower tendency to attach onto the surface; consequently, the flux through the modified membrane declined only slightly from 90 ml min^−1^ bar^−1^ to 65 ml min^−1^ bar^−1^ after five cycles, that is about 27%. The results indicate that the microorganisms tended to foul fast on the pristine membrane surface without chitosan layers.

This reveals that the chitosan coating is an economical and effective layer against bacteria adsorption, which could make the membrane cleaning cycles more efficient without a drop in the operating flux. The hydrophilic moieties on chitosan strongly interact with water molecules by hydrogen bonding and provide a hydration layer on the surface of the coated membrane, as an effective and inexpensive barrier to deter the bacteria (*E. coli*, *Salmonella* and *Legionella*) from directly attaching onto the membrane surface. Additionally, these membranes have a high removal efficiency for genetic material from water. Amyloid fibrils play a crucial role in the adsorption of the genetic material. The membrane can be reused for several cycles without dropping in any efficiency. Additionally, the hybrid membrane surface can be modified by a chitosan coating film to improve the anti-biofouling properties. This surface modification improved the reduction of the bacteria rejection by 2 log and also retained the flow rate and increased the anti-biofouling properties. These results indicate that AHMs have great potential in the simultaneous and efficient removal of bacteria and genetic material from contaminated water sources.

## 3. Conclusions

Self-standing amyloid hybrid membranes (AHMs) are prepared by the direct addition of amyloid and activated carbon to the cellulose pulp solution. The mesh size of these AHM can easily be tuned or adjusted by the membrane preparation protocol, which can effectively remove the bacteria (*E. coli*, *Salmonella* and *Legionella*). Additionally, these membranes have a high removal efficiency for genetic material from water. Amyloid fibrils play a crucial role in the adsorption of the genetic material possibly due to hydrogen bonding, as well as hydrophobic, van der Waals and electrostatic interactions among the positively charged groups of the ampholytic amyloids and the negatively charged nucleic acids. The membrane can be reused for several cycles without dropping in any efficiency. Additionally, the hybrid membrane surface can be modified by a chitosan coating film to improve the anti-biofouling properties. This surface modification improved the reduction of the bacteria rejection by 2 log and also retained the flow rate and increased the anti-biofouling properties. These results indicate that AHMs have great potential in the simultaneous and efficient removal of the bacteria and genetic material from contaminated water sources.

## Conflicts of interest

S. B. and R. M. are inventors of a patent filed on ETH behalf.

## Supplementary Material

NA-002-D0NA00189A-s001
