# Supplementary material for: Amyloid hybrid membranes for bacterial & genetic material removal from water and their anti-biofouling properties
Source: Nanoscale Adv. 2020 Sep 11;2(10):4665–70. doi: 10.1039/d0na00189a (PMC9419293; doi:10.1039/d0na00189a)
Supplement: NA-002-D0NA00189A-s001 [file NA-002-D0NA00189A-s001.pdf]

Electronic Supplementary Material (ESI) for Nanoscale Advances.
This journal is © The Royal Society of Chemistry 2020

Electronic Supplementary Information (ESI)

Amyloid Hybrid Membranes for Bacterial & Genetic Material Removal from Water and their Anti-biofouling Properties

Archana Palika, Akram Rahimi, Sreenath Bolisetty, Stephan Handschin, Peter Fischer, Raffaele Mezzenga

Table of contents

1. Supporting Materials and Methods 1-8
2. Supporting Figures 9-13
3. Supporting references 14

1. Supporting Materials and Methods

Materials: β-Lactoglobulin (BLG) protein was purified from whey protein isolate received as a kind gift from Fonterra, New Zealand. Cellulose was purchased from Khadi papers. Activated carbon (Norit SAE Super) was purchased from Cabot. Giluton (polyamidoamine-epichlorohydrin) was used as a wet strengthening agent, purchased from Kurita paper solutions technologies. *E. coli* K12 bacteria, *Salmonella* DT7155, *Legionella pneumophila* DSM 7513 were provided by the Martin Loessner group, ETH Zurich. BCYE (Buffered charcoal yeast extract) agar plates were purchased from Thermo Fisher Scientific. BYE (Buffered yeast extract medium) broth was prepared by mixing of 10 g/L yeast extract, 2.2 g/L potassium hydroxide (KOH), 1 g/L α-ketoglutarate, 0.4 g/L L-cysteine, 10 ml/L of Iron supplement (0.25 g ferric pyrophosphate dissolved in 10 ml Milli-Q water and filter sterilized). LB (Lysogeny Broth) was prepared by mixing the 10g/L of tryptone, 5g/L of yeast extract, 5 g/L of NaCl, PBS (Phosphate buffered Saline) was prepared by mixing 8 g/L NaCl, 0.2 g/L KCl, 1.44 g/L Na_2HPO_4, 0.24 g/L KH_2PO_4. Chitosan was purchased from Sigma-Aldrich.

Purification of β-Lactoglobulin (BLG) Monomers

The purification of *β*-lactoglobulin and the preparation of amyloid fibrils from the purified protein are discussed in our earlier reports. Briefly, 10 wt% whey protein powder was dissolved in Milli-Q water and the pH was adjusted to 4.6 and centrifuged at 15000 rpm for 15 min. The supernatant was collected in a clean bottle and the pH adjusted to 2 by using a 2 M HCl solution and dialyzed using a dialysis membrane with 6-8 kDa cutoff against Milli-Q water at 4°C for 5 days. Finally, the dialyzed solution was filtered through the 0.22 μm cellulose acetate membrane filter, freeze-

dried into powder and stored at -20°C. Amyloid fibrils were prepared by heat denaturation of the 2 wt% monomer solution at pH 2 at 90°C for 5 hours.

Preparation of amyloid hybrid membranes (AHM)

AHMs were prepared by adding different proportions of cellulose pulp, carbon and amyloid respectively. Giluton was added as a wet strengthening agent to the above mixture. To prepare a 4 cm diameter and 2 mm thickness membrane, 6.6 g of cellulose paper was added to the 1300 ml of tap water and soaked for 15 min. This mixture was then ground for 2 min to prepare a homogenous mixture of cellulose fibers, followed by the addition of 4 g of activated carbon, 11 ml of amyloid fibrils (2 wt% solution) and 325 μl of giluton. The above mixture was passed through a sieve having a mesh size of 0.35 mm by slow pressing. The membrane with 2 wt% amyloid fibrils composition was finally dried in an oven at 90°C overnight.

To investigate the relationship between pore size, filtration efficiency, and flux, Cellulose fibrils were ground. The increase in grinding time resulted in the shortening of the fibril length. Additionally the membranes were pressed to remove extra water. We observed that the decreased pore size and flow rate increased the bacterial removal efficiency from 99.565% to 99.99%.

Table S1 – Relationship between separation performance, flow and average pore size

Flow rate	Log reduction	Average pore size	Cellulose grinding time
19ml/min	99.565%	1.2 µm	20 secs
16.8ml/min	99.965%	1 µm	40 secs
14ml/min	99.977%	800 nm	60 secs
5ml/min	99.99%	400 nm	2 mins

Specific Genetic Material Adsorption Capacities of Individual Components of AHM

To determine the specific adsorption capacity of each component of AHM, 3 membranes were prepared separately using the above procedure. One was with cellulose, the second one with cellulose and carbon and the third one was with cellulose, carbon and amyloid (AHM). 10 ml of genetic material with the concentration of 14.2 ppm was passed through these membranes and the adsorption capacities of carbon, cellulose and amyloid were determined.

Preparation of E. coli and Salmonella Cultures

Initially, bacterial cultures were revived from -20 °C by streaking on an LB agar plate and kept overnight at 37°C. A single colony from each of the bacterial strains was freshly grown in 6 ml of LB medium and incubated at 37 °C. The optical density of the cells was measured using a Biochrom Libra S22 UV-Vis spectrophotometer. Cells were harvested by centrifugation under specific conditions (6000 rpm, 10 min) and cell pellets of Salmonella and *E. coli* were suspended in 5 ml

of PBS and then diluted with 45 ml PBS (pH 7) respectively. Filtration tests were performed using these bacterial solutions.

Preparation of Legionella Cultures

Legionella cultures from -20 °C were grown on BCYE agar plates for 3 to 4 days at 37 °C. A single colony from an agar plate is inoculated into 6 ml of BYE medium and grown at 37 °C. The optical density of the bacterial culture was measured after 3 to 4 days of incubation. Cells were harvested by centrifugation under specific conditions (6000 rpm, 10 min) and the cell pellet was suspended in 5 ml of PBS and then diluted with 45 ml of PBS. The resulting solution was used for filtration experiments.

Serial dilution method to detect the concentration of bacterial cultures

Serial dilution is the simplest technique for determining concentrations of bacterial cultures. A 100 µl of bacterial culture is added into an Eppendorf tube that contains 900 µl of Phosphate buffered saline. This process is repeated by aliquoting 100 µl of the newly created solution and adding it into the next tube. Aliquoting and resuspension continue in this way, diluting the bacterial culture concentration by a factor of 10 with each step[1]. Then the dilutions were plated on respective plates and incubated at 37 ºC overnight. The colonies that grow on the agar plate arise from a single cell and each colony can be counted to estimate the number of colony-forming units per milliliter (CFU) and represented as CFU/mL.

$$Bacterial\ Concentration \frac{CFU}{ml} = \frac{Dilution\ factor \times Average\ no\ of\ colonies}{Volume\ of\ diluted\ bacteria\ added\ to\ the\ medium}$$

Preparation and measurement of the concentration of Genetic Material

50 ml of *E. coli* bacterial cultures were prepared from the above procedure and sonicated in total for 10 min. Sonication is the physical approach commonly used to disrupt the cellular membranes and release the cell contents. The method uses high-frequency sound waves to agitate and lyse cells, bacteria and spores. To prevent excessive heating, ultrasonic treatment was applied in multiple short bursts to a sample immersed in an ice bath. The 50 ml were split into portions of 10 ml of bacterial cells which were filled in falcon tubes and the resonance frequency of 19-21 kHz was applied for 5 min. Sonication was repeated for 2 cycles, each cycle for 5 min. Sonicated *E. coli* cells were filtered through 0.44 μm cellulose acetate filters to remove bacterial debris and residual bacterial cells. The concentration of the genetic material was measured using an absorption-based Nanodrop (Thermo Scientific Nano Drop 2000)

Filtration Experiments

45 ml of each bacterial culture (*E. coli*, *Salmonella* and *Legionella*) was introduced aseptically into a sterile cell filtration system containing the AHM. The filtration system was operated under negative pressure by using a vacuum pump. To detect the concentration of the bacterial cultures before and after the filtration, serial dilutions were performed, plated on respective agar plates and incubated at 37 °C. Colony-forming units were measured to determine the CFU/ml. This experiment was done in triplicates for *E. coli*, *Salmonella* and *Legionella*. Similarly, 10 ml of genetic material was filtered through AHM and also separately through cellulose, carbon and cellulose membranes. The concentration of genetic material before and after filtration was measured by using NanoDrop.

Surface Modification of the AHM

The surface coating of the AHM was performed by immersing the membrane into the chitosan solution. Firstly, 1 w/v % chitosan solution was prepared in a 2 wt% aqueous acetic acid solution under constant stirring for 3 hours at room temperature. The pre-wetted hybrid membrane was immersed in the chitosan solution for 5 min. The chitosan-coated membrane was then placed in an oven at 50º C for 3 hours to vaporize the solvent from the coated layer completely. After that, the coated membrane was rinsed with NaOH solution (0.1 M in 50 v/v % water/ethanol mixture) for few hours to neutralize the chitosan film, followed by thorough washing with 50 %v/v ethanol for 10 min and distilled water for 30 min. Finally, the membrane was dried at room temperature.

Anti-biofouling Performance

The anti-biofouling behavior of the pristine and coated AHM was evaluated by a cyclic filtration experiment, in which the *E. coli* K12 strain was used as a model biofoulant. A cell filtration system with suction pressure and volume capacity of 100 ml was used to characterize the filtration performance of the prepared AHM. All supplies were sterilized in an autoclave before the filtration. The changes of the flux during pure water and during bacteria solution filtration were recorded to estimate the biofouling progress, which was carried out in three steps: 1) the pure water flux of the membrane was quantified; 2) the membrane was exposed to the bacteria solution for 2 hours and then, the flux for the bacteria solution was measured; 3) the fouled membrane was immersed in fresh PBS to remove free bacteria for 10 min, and then, the second pure water flux of the washed membrane was measured again, indicating the end of one filtration cycle. The flux per pressure drop in all the steps, defined as *J*, was calculated by the following equation:

$$J = \frac{V}{t \times P} = \frac{kA}{\mu L} \tag{1}$$

where J (ml min^{-1} bar^{-1}) is the flux per pressure drop over a period of filtration time t (min), V (ml) refers to the collected volume of the permeated liquid, P represents the applied pressure (or pressure drop in Darcy's law), k is the membrane permeability, A the membrane area, μ is the viscosity of the solution and L the thickness of the membrane.

For determining the bacteria rejection of pristine and modified membranes, permeate bacterial solution was serial diluted and plated on LB agar plates. After overnight incubation bacteria colonies were counted. The membrane bacteria rejection was measured by the following equation:

$$R = \frac{Nf - Np}{Nf} \times 100 \qquad (2)$$

Where N_f and N_p are the numbers of live bacteria in the feed solution and permeate solution, respectively.

Scanning Electron Microscopy (SEM)

For SEM imaging pieces of the filter were cut with a blade and fixed on SEM aluminum stubs with conductive carbon (Plano GmbH, Germany). After drying, the samples were sputter-coated with 4 nm of platinum/palladium in a CCU-10 sputter-coater (Safematic, Switzerland). Inlens and secondary electron images were recorded at 2 kV with a Zeiss Leo 1530 FE-SEM (Zeiss, Germany).

Transmission Electron Microscopy (TEM)

For TEM imaging 5µl of a 0.1wt% dispersion of BLG-fibrils were spread onto a glow discharged (Emitech K100X, Great Britain) carbon-coated Cu grid (Quantifoil, Germany). After 1 min, excess liquid was drained off with filter paper, washed twice with double distilled water and 5µl of a 2% aqueous uranyl acetate solution was placed on the grid for 1s followed by a second step with 5µl uranyl acetate for 15s. The dried specimens were examined with a TEM Morgagni 268 (Thermo Fisher Scientific, USA) operated at 100kV.

2. Supporting Figures

S1 Adsorption isotherms

Adsorption isotherms of the membrane were determined by filtering genetic material through a 0.0002 m2 AHM membrane and the concentration of the adsorbed genetic material after each cycle is measured by using a Nano Drop spectrophotometer. From the adsorption isotherm, 1200 g of the hybrid membrane can remove 124 g of genetic material**.**

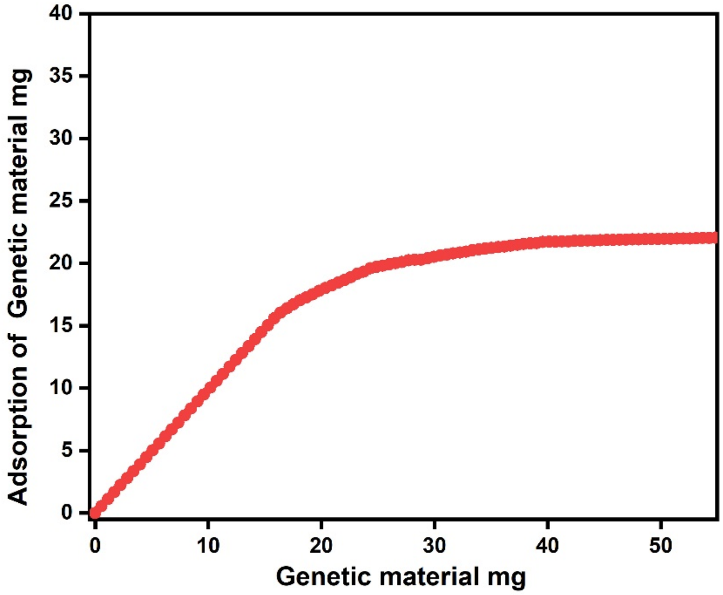

Figure S1. Adsorption isotherm for the genetic material removal by the AHM.

S2 Anti-biofouling

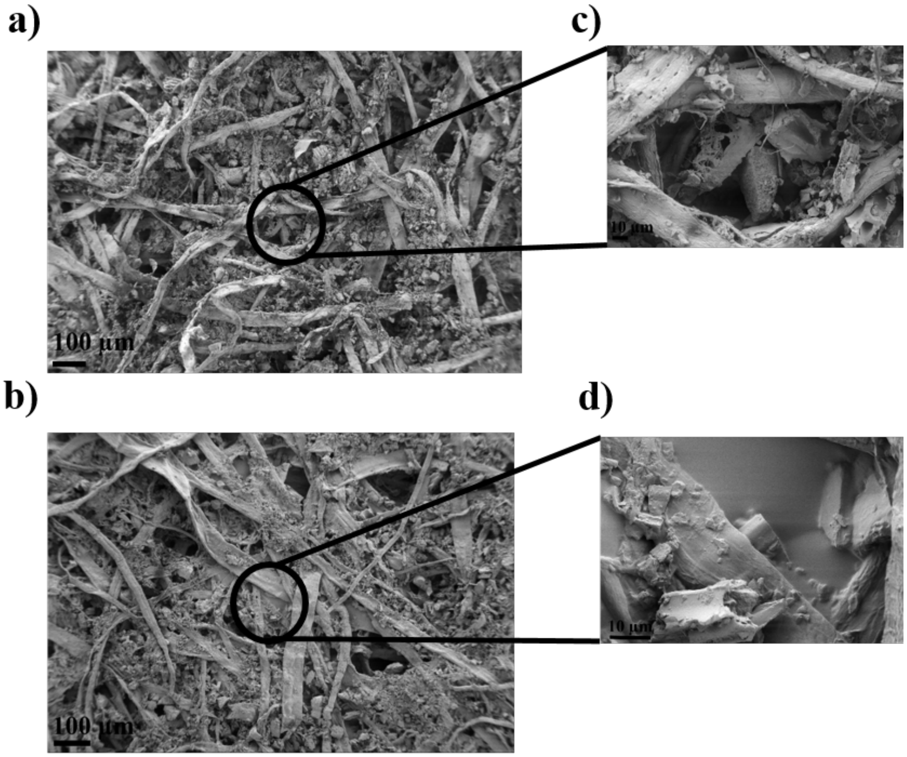

Figure S2. a) SEM image of the surface morphology of unmodified AHM, b) SEM image of CCAHM, c) Enlarged section of the SEM image of unmodified AHM and d) Enlarged section of the SEM image of CCAHM. The cellulose fiber branching, change in width and kinks along the contour length arise from the assembly of individual nanofibrils, leading to an amplification of defects. For a detailed discussion on the structure of nanocellulose nanofibrils the reader is addressed to earlier reports [2].

S3 ***Poresize of AHM measured with POROLUX 100***

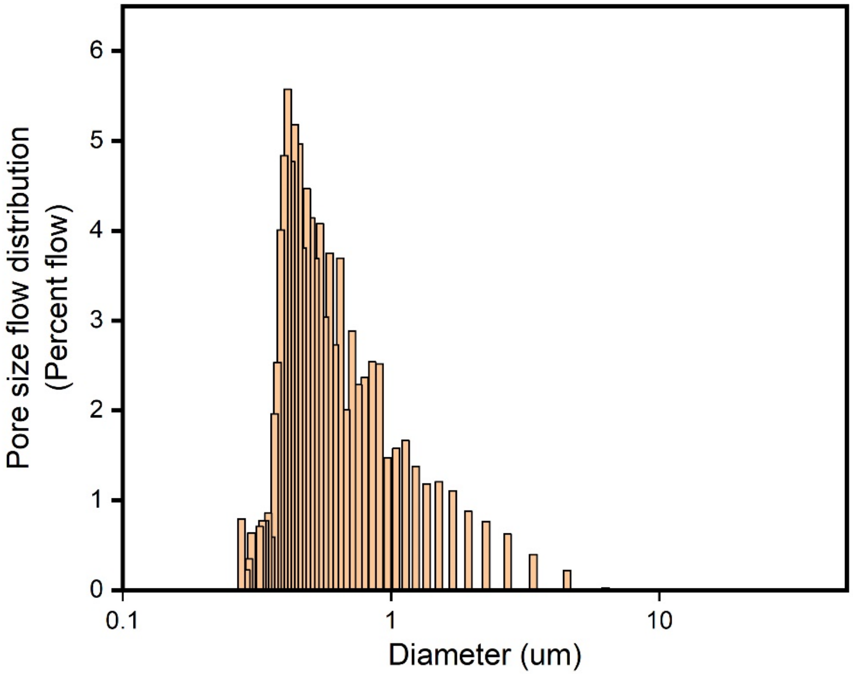

Figure S3 *Poresize of AHM measured with POROLUX 100*

S4. Fourier transform infrared (FT-IR) spectra of pure chitosan, AHM and CCAHM

To confirm the presence of chitosan and the chemical composition of the top surface of membranes, the pure chitosan and the top surfaces of AHM and CAHMs were characterized by FTIR. Pure chitosan shows the characteristic peaks at 1621 cm–1 and 1544 cm–1 (amide I and II functional groups)[3,4]. The data analysis of FTIR reveals that AHM does not show any characteristic peaks of Chitosan whereas the CCAHM membrane shows the characteristic peaks at 1621 cm–1 and 1544 cm–1 indicating successful coating of chitosan on to the membrane

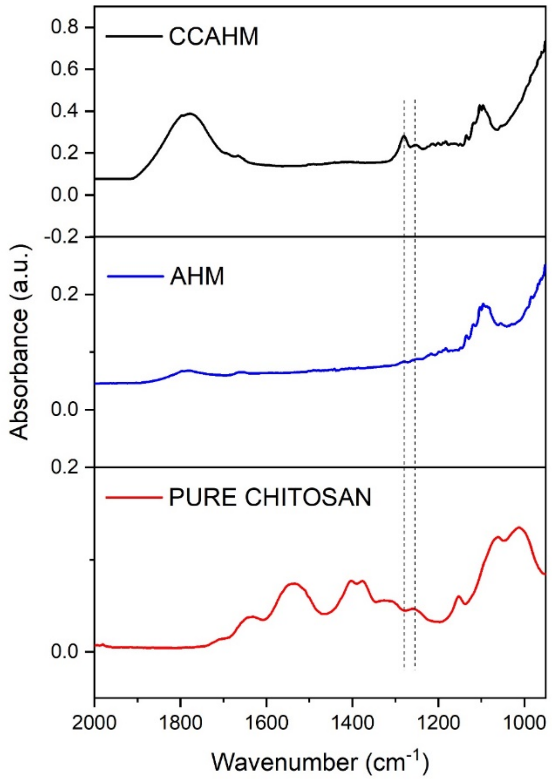

Figure S4 *FTIR spectra of CCAHM,AHM and pure chitosan*

Contact angle measurements

Contact angles of both CCAHM and AHM were studied. The direct contact angle measurements of the AHM was not possible to be measured as the membranes are highly porous and the water droplet adsorbs immediately on contact.

The hydrophilicity of the Chitosan membrane was studied by contact angle measurements. Theoretically, the surface having contact angle lower than 90° is considered as hydrophilic. Water droplet was placed on the membrane surface and pictures were taken to measure both receding contact angle and advanced contact angles. For chitosan membranes, the receding contact angle is

0° and advance contact angle is 75 ° therefore the contact angle lays between 0° to 75°, indicating an hydrophilic surface.

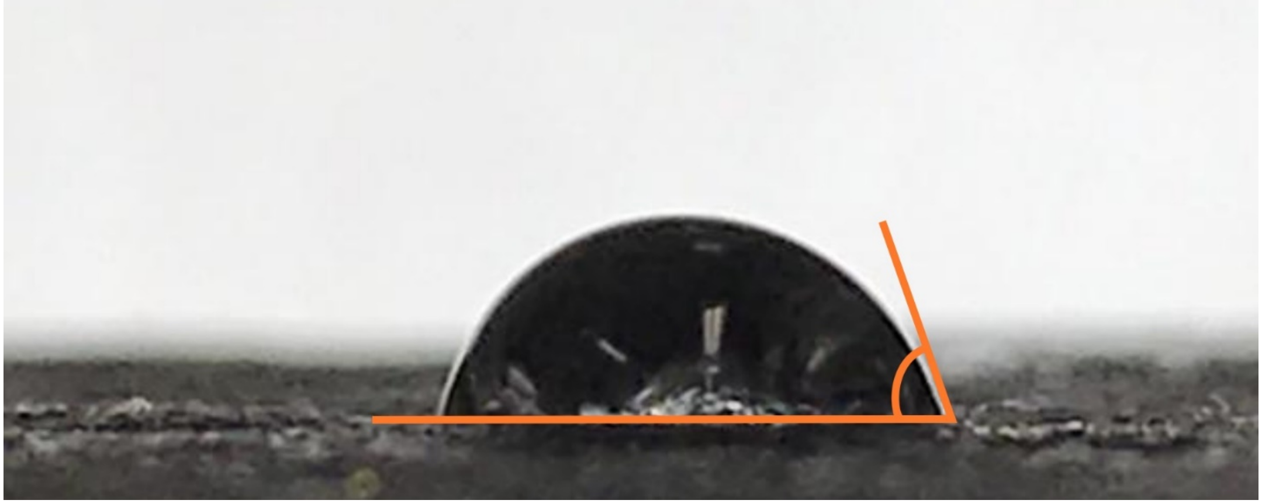

Fig S5 Advance Contact angle of Chitosan membrane

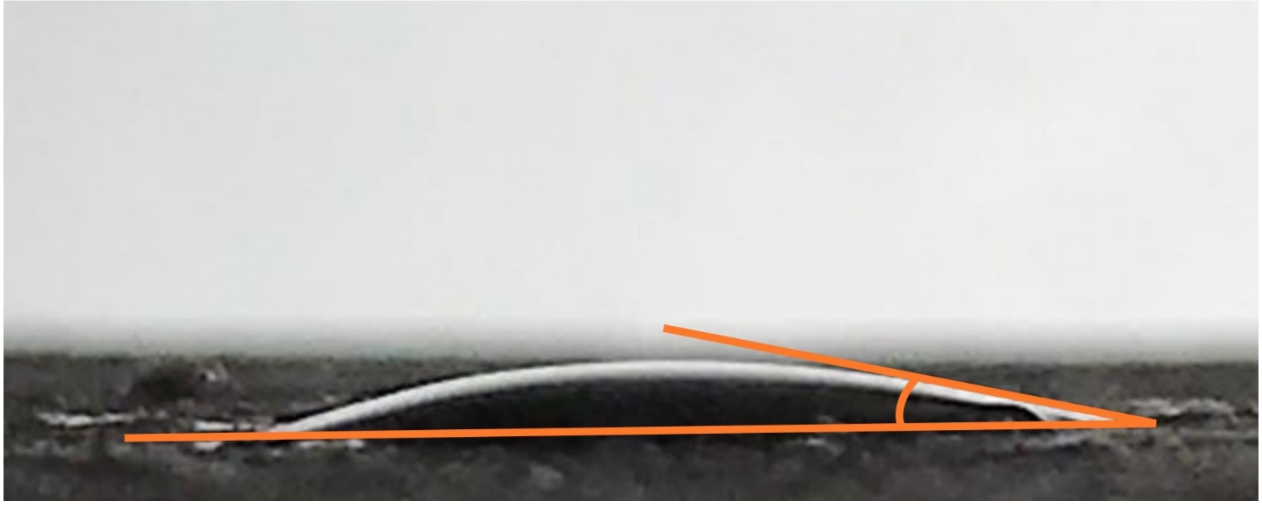

Fig S6 Receding contact angle of Chitosan membrane

References

1, M. R. Sadzikowski, J. F. Sperry and T. D. Wilkins, *Appl. Environ. Microbiol.*, 1977, **34**, 355–362.

2. Usov, G. Nyström, J. Adamcik, S. Handschin, C. Schütz, A. Fall, L. Bergström and R. Mezzenga, *Nat. Commun*., 2015, **6**, 7564.

3. M. Purwanto, L. Atmaja, M. A. Mohamed, M. T. Salleh, J. Jaafar, A. F. Ismail, M. Santoso and N. Widiastuti, *RSC Adv.*, 2016, **6**, 2314–2322.

4. M. Mathaba and M. O. Daramola, *Membranes*, 2020, **10**(3): 52.
